# Adult and adolescent livestock productive asset transfer programmes to improve mental health, economic stability and family and community relationships in rural South Kivu Province, Democratic Republic of Congo: a protocol of a randomised controlled trial

**DOI:** 10.1136/bmjopen-2016-013612

**Published:** 2017-03-14

**Authors:** Anjalee Kohli, Nancy A Perrin, Mitima Mpanano Remy, Mirindi Bacikenge Alfred, Kajabika Binkurhorhwa Arsene, Mwinja Bufole Nadine, Banyewesize Jean Heri, Mitima Murhula Clovis, Nancy Glass

**Affiliations:** 1 Johns Hopkins University School of Nursing, Baltimore, Maryland, USA; 2 Center for Health Research, Portland, Oregon, USA; 3 Programme d'Appui aux Initiatives Economiques (PAIDEK), Bukavu, Democratic Republic of Congo; 4 Pigs for Peace, Bukavu, Democratic Republic of Congo

**Keywords:** MENTAL HEALTH, impact evaluation, Conflict, Microfinance, Democratic Republic of Congo

## Abstract

**Introduction:**

People living in poverty have limited access to traditional financial institutions. Microfinance programmes are designed to meet this gap and show promise in improving income, economic productivity and health. Our Congolese–US community academic research partnership developed two livestock productive asset transfer programmes, Pigs for Peace (PFP) and Rabbits for Resilience (RFR), to address the interlinked health, social and economic well-being of individuals, their families and communities. The community-based randomised controlled trials examine the effectiveness of PFP and RFR to improve health, economic stability, and family and community relationships among male and female adults and adolescents living in 10 rural, postconflict villages of eastern Democratic Republic of Congo.

**Methods and analysis:**

PFP participants include adult permanent residents of rural villages; adolescent participants in RFR include male and female adolescents 10–15 years old living in the selected rural villages. Participants were randomised to intervention or delayed control group. Participants in PFP completed baseline interview prior to intervention and follow-up interview at 6, 12 and 18 months postintervention. In RFR, participants completed baseline interview prior to intervention and follow-up interview at 6, 12 and 18 months postbaseline. The primary outcome of both trials, the change in baseline mental health distress at 18 months in the intervention group (adults, adolescents) compared to control group, is used to calculate sample size.

**Ethics and dissemination:**

The Johns Hopkins Medical Institute Internal Review Board approved this protocol. A committee of respected Congolese educators and community members (due to lack of local ethics review board) approved the study. The findings will provide important information on the potential for community-led sustainable development initiatives to build on traditional livelihood (livestock raising, agriculture) to have a sustained health, economic and social impact on the individual, family and community.

**Trial registration number:**

NCT02008708, NCT02008695.

**Strengths and limitations of this study**

- This study provides important information on the implementation of rigorous intervention research in postconflict, low-resource settings. Findings from this study provide essential information on effective and sustainable interventions to improve health, economic stability and relationships in rural, postconflict communities.
- The Johns Hopkins—Programme d'Appui aux Initiatives Economiques du Kivu partnership is strong and collaborative; as a result, the research strategies and tools closely match programme objectives and were well adapted to the participant experiences.
- The Pigs for Peace and Rabbits for Resilience datasets contain rich, longitudinal information on households including parents and their children. This important information fills a gap in global data on the bidirectional relationship between parent/caregiver health and well-being and adolescent health and well-being.
- Randomised recruitment of participants took place at the individual level, and not the village level, to allow participants to self-select to participate. Therefore, findings provide key information on the individual level, but cannot be extrapolated to the village level.
- Despite multiple efforts to ensure that adult and adolescent participants understood the microfinance-related commitments to programme participation, some adults and adolescents may have self-selected to participate in the programme expecting the loans to be (or become) a gift or donation as many humanitarian organisations in the area have used the donation model of programming.

## Introduction

Seventy per cent of the global poor reside in rural areas where they largely depend on agriculture and livestock for income.^1^ Globally, people living in poverty have limited access to traditional financial institutions. In Sub-Saharan Africa, formal banking institutions have not yet extended their services across communities aside from selected urban centers. Access to services from these established urban financial institutions including credit and savings is often limited to upper and middle-class Africans who can demonstrate collateral, economic security and have knowledge of and access to banking services.^2^ As a result, urban and rural poor remain marginalised from banking services leaving the majority of the population to rely on local cooperative groups, money lenders, family and friends in times of urgency, to cover regular expenses and to initiate or grow small businesses. Extreme poverty, as defined by the World Bank, includes populations that live under $1.90 a day (based on 2011 US$ exchange rate).^3^ Owing to their irregular access to income or self-exclusion because of concerns about repaying loans, the extreme poor may be excluded from local lending systems including cooperative groups.^4^

Poverty and health are closely related. Ill health may increase poverty and debt due to medical expenses and loss of work.^5^ ^6^ Poor families are often unable to support the cost of healthcare, even when necessary, as prices are burdensome when salaries are low and irregular.^7^ Microfinance programmes, when well-crafted to the target population, show promise in improving income, economic productivity and health while maintaining reasonable repayment rates. A review of integrated microfinance programmes demonstrate their potential for financial and health benefits including increases in women's empowerment and decision-making,^8^ reductions in risk of physical and sexual intimate partner violence (IPV),^8^ ^9^ increases in health knowledge and use of health services.^7^ Microfinance programmes that are built on local knowledge are more likely to be acceptable, sustainable and effective.^10^ Poor, rural households in developing countries are often engaged in livestock production with the livestock asset acting as a safety net for the family and supporting health including food consumption and emergency needs.^11^ Recent research shows the potential of gender transformative livestock asset transfer programmes (including conditional livestock asset transfer) to increase household dietary diversity and food security,^12^ ^13^ improve mental health,^13^ delay adolescent girls age of marriage and increase their educational achievements.^14^ Livestock asset transfer programmes and other microcredit programmes may act directly to improve mental health or indirectly through reducing frequency of illness, fewer days missed from work due to sickness, reduced IPV, improved food security and nutrition for self and family.^9^ ^15–17^ More research is needed to understand the effectiveness of livestock asset transfer programmes on mental health, household economic stability, the sustainability of intervention impact and the effect of individual intervention components including on women's empowerment, decision-making and household dynamics. This research is especially important for postconflict and humanitarian settings where civilians have lost social and economic support structures and been exposed to events that may result in mental distress.

Studies in conflict and postconflict settings show a cyclical relationship between conflict and poverty. Conflict, through destruction of institutions and infrastructure, social services, displacement, loss of resources and assets and injury negatively impacts economic growth and productivity.^18^ War creates informal economies that enable opportunistic behaviour, economic activities focused on short-term rewards and segmented markets. Rural populations bear the heaviest consequences of conflict through loss of livestock and land, crop and infrastructure destruction, forced displacement and inability to access markets.^19^

The Democratic Republic of Congo (DRC) is an example of the way prolonged conflict exacerbates poverty and poor health with 87.7% of the Congolese population living in extreme poverty as measured by daily income (<$1.25/day).^20^ With 50% of men and women ages 15–59 years working in agriculture,^20^ the challenges to alleviating poverty and building sustainable economic opportunity in the DRC are great. Decades of oppression and corruption during the colonial period and the 30-year despotic reign of Mobutu Sese Seko (1965–1997) resulted in an under-educated population with limited access to economic resources, employment and basic health and social services.^21^ Further, nearly two decades of conflict in Eastern DRC following the collapse of the Mobutu government and genocide in neighbouring Rwanda has resulted in an estimated 6 million premature deaths.^22^ In addition to witnessing and experiencing traumatic events, families in rural areas report pillaging of household resources including sources of economic livelihood (eg, livestock/animals, land), the destruction of education and health facilities and family and community level trauma.^23^ ^24^ However, in the context of these adversities, Congolese demonstrate resilience in rebuilding their futures, for example, through active participation in locally led microfinance programmes. Economic interventions implemented in conflict-affected rural areas must account for the lack of income opportunities and access to traditional forms of credit, limited health and social support infrastructure as well as trauma and loss that many individuals and families have endured. These challenges make it essential to engage with communities in developing interventions and address the multiple and inter-related economic, health and social conditions of rural families to achieve sustainable improvements. Therefore, our Congolese-US community-academic research partnership between Programme d'Appui aux Initiatives Economiques du Kivu (PAIDEK) and Johns Hopkins University School of Nursing was initiated in 2008. PAIDEK is an established Congolese microfinance organisation working throughout eastern DRC since 1996. The partnership led to the development of two livestock microfinance programmes, Pigs for Peace (PFP)^17^ ^25^ and Rabbits for Resilience (RFR) that address the interlinked health, social and economic well-being of individuals, their families and communities. Our manuscript details the design of two community-based randomised controlled trials to assess the impact of our livestock/animal microfinance interventions, PFP: Microfinance Intervention to Improve Health of Trauma Survivors in DRC (NCT02008708) and RFR: Youth and Adult Microfinance to Improve Resilience Outcomes in Democratic Republic of Congo (NCT02008695) to improve economic stability, health and family relationships among rural poor in postconflict settings.

### Overview of community-led livestock programmes: PFP and RFR

The experience of our Congolese partner, PAIDEK, indicated that rural villagers would be hesitant to take cash loans because of their significant and multiple financial needs and their limited potential for repayment of the loans plus interest given the considerable loss of wealth and livestock due to looting during the wars and the lack of income generating activities in rural villages. The trauma of prolonged conflict and violence limited productivity of household members as women and men fear for their safety when working outside the home on farms or travelling to sell goods in nearby markets or mines. Through multiple discussions with area administrators and village leaders, livestock productive asset transfer was determined as the potential way forward to rebuild economic security, health and relationships in rural areas.

The livestock productive asset transfer model builds on traditional forms of livelihood and wealth. Farming and breeding animals (ie, cows, goats, pigs, chickens) is a primary source of food and income for rural households in the 10 participating rural villages. Pigs are common to the area and do not need large amounts of space to live and forage, reducing the need for people to travel far from the villages to seek food and exercise pigs. Pigs eat everything that humans can eat so they do not need special feed and can reproduce twice annually, averaging six piglets per breeding. Importantly, whereas men in the DRC usually control household decisions regarding the sale of goats or the family cow as they are tied to the dowry system, women and men can make decisions about the purchase, breeding and selling of the family pig. Therefore, engaging women and men as the primary loan recipient for a pig would not create conflict in the household when the loan recipient made decisions such as reimbursing the project, breeding and selling the animal for funds to pay for healthcare, school fees or other family needs. A piglet (3 months of age) can be sold at $25–$30 in the area markets, a significant supplement to the estimated gross national income (2011 Purchasing Power Parity) of US$680 annually.^20^ Rabbits were chosen for the adolescent-led microfinance RFR programme for many of the same reasons noted above, specifically, rabbits can live in contained spaces, eats food that is available within the rural village and is a common animal for a boy and girl to raise, breed, eat and sell. Adolescents can sell a rabbit for $5–$8 to help supplement funds for education, healthcare and nutrition.

PFP and RFR are village-based interventions designed to improve the multiple and inter-related economic, health and relationships of rural individuals and families. In PFP, adult men and women participate in microfinance training; gain support by our skilled microfinance team; agree to build a pigpen for their 2–4-month old female pig loan and compost for pig waste as per project standards. Further, participants agree to actively engage with other PFP participants in community meetings, provide adequate care for their pig and reimburse the project 2 female piglets when their pig loan gives birth, one piglet to repay the loan and one piglet for interest on the loan. These repayment piglets are then given to other PFP members often in the same community as a loan. The original female pig loan and the remaining piglets are for the PFP household to continue to raise, breed and sell with continued support of the PFP microfinance team. RFR microfinance was developed with similar principles to the PFP microfinance intervention. The project works primarily with young male and female adolescents (10–15 years of age) that are interested and committed to raising rabbits with the support of their parent/caregiver, including building a rabbit cage, providing care for the rabbit, participating in a training programme and community meetings with support by our skilled RFR team and repaying the loan to the project in the form of two female rabbits when the original rabbit gives birth. These rabbit loan repayments are then given to other adolescents in the project. Similar to PFP the original rabbit and remaining offspring are for the RFR members to continue to raise, breed, eat and sell as decided by the adolescent in collaboration with their parent/caregiver. Facilitated monthly community meetings and home visits with both PFP and RFR members are conducted to discuss challenges and identify solutions related to participation, guide new members in raising livestock/animals and encourage timely repayment of loans. Our Congolese team members facilitate regular visits by a project veterinarian technician to review animal care and vaccination.

Despite efforts to engage local leadership, explain programme principles to interested participants (including the obligation of loan repayment) and provide healthcare to loan animals and training for participants, some adult or adolescent participants may not be successful in the programme. If the loan animal dies prior to their first cycle of birth, Congolese team members evaluates the participant's dedication to the programme (eg, maintained a clean animal cage, ensured the nutrition and health of the animal) based on prior home visits in making decisions to replace a loan animal. As some animals may have been stressed by transport or sudden illness, dedicated and committed PFP and RFR participants are given a replacement loan animal. Participants who are eager to reinvest in the programme but had not properly cared for their loan animal are encouraged to purchase a replacement animal with their own money. If they replace their loan animal, our programme continues to provide support and guidance to the participant. In community meetings led by PFP participants and Congolese team members, defaulters whose animal loan die due to negligence are instructed to repay the programme the cost of the animal loan.

Our Congolese–US partnership was initiated in December 2008 with the development and implementation of the PFP demonstration project with 10 families in one village in South Kivu province of Eastern DRC. Over the next 2 years, the PFP demonstration project expanded and through a cross-sectional evaluation with the first 100 participants, we recognised the potential health, social and economic benefits of the livestock/animal asset.^25^ For example, the vast majority (92%) of participants in the demonstration project said that their relationship with the community had improved. They also reported using the money from selling the pigs for food, school fees, housing improvements, healthcare fees and expansion of livestock or other small businesses. After reviewing these findings, our collaboration decided to continue implementation and expansion and prioritised a rigorous evaluation of the Congolese-led microfinance programmes in 10 rural villages to improve the economic stability, health and relationships of individuals, families and communities.

### Study aims

Two community longitudinal randomised controlled trials are being conducted to evaluate the effectiveness of the PFP (2010–2016) and RFR (2012–2017) interventions to improve household economic stability, health and relationships among adults and young adolescents living in 10 rural, postconflict villages in South Kivu Province of Eastern DRC. The RFR impact evaluation assesses the effectiveness of a youth-led programme, RFR, combined with the adult microfinance, PFP, programme on youth, family and community resilience outcomes. The study aims are detailed in table 1; primary and secondary outcomes are described later in this manuscript.

**Table 1:** PFP and RFR study design

	Pigs for Peace evaluation	Rabbits for Resilience evaluation
Research aims	Determine the effectiveness of village-led productive asset transfer (PFP) on: Health and reintegration; Household economic stability; and Village-level health, economics, stigma and reintegration compared to delayed control groups.	Compared to adolescent-led productive asset transfer only and PFP only approaches, determine the relative effectiveness of adolescent-led productive asset transfer combined with PFP on: Adolescent resilience (school attendance, relationship with family members and peers, health, self-esteem, outlook for the future); Family resilience (household economic stability, food security, parent/caregiver health); and Community resilience (youth and adult engagement in activities).
Selection of villages	Ten rural villages of Walungu and Kabare Territory, South Kivu province based on: Feasibility of delivering an intervention over a wide geographic area; Commitment to the intervention and study by traditional chiefs and administrators; Findings from village level assessments	The same villages as in the PFP impact evaluation
Participant eligibility criteria	Men and women, 16 years and older that: Expressed an understanding of and commitment to microfinance principles Were permanent residents of the village Were responsible individuals in the household (eg, married 16-year-old, 16-year-old responsible for younger siblings because of death of parent)	Male and female adolescents between 10–15 years old that: Expressed an understanding of and commitment to microfinance principles; Were permanent residents of the village; and their parents/caregivers

PFP, Pigs for Peace; RFR, Rabbits for Resilience.

## Methods and analysis

### Study location

**Figure 1:**
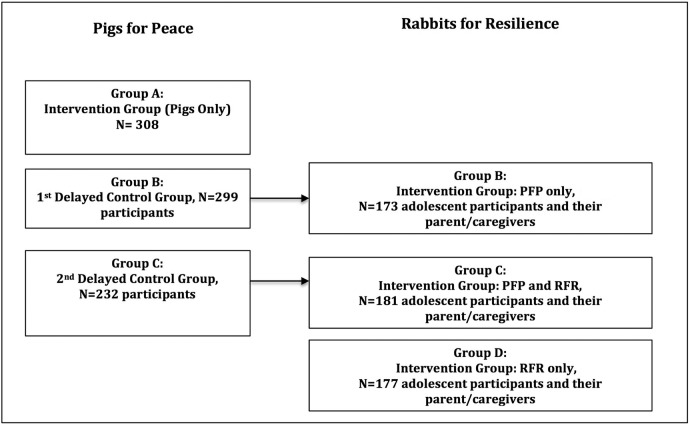
PFP and RFR intervention and delayed control groups in all 10 participating villages of Walungu and Kabare Territories. PFP, Pigs for Peace; RFR, Rabbits for Resilience.

Ten villages in Walungu and Kabare Territories in the tribal chiefdom of Ngweshe located in South Kivu Province of eastern DRC were selected for participation in the PFP and RFR impact evaluations. The chiefdom is about 40–80 km south of Bukavu, the capital city of South Kivu Province and has an estimated population of 700 000 residents. The Territories were chosen for several reasons: (1) this rural area suffered significantly from conflict because of its proximity to mineral resources (eg, gold, coltan) and isolation due to limited infrastructure; (2) very limited humanitarian and/or development resources including microfinance, have reached the Chiefdom; and (3) the team has a strong history of respect and collaboration (since 2007) with local leaders (physicians, nurses, agricultural workers, village leaders, etc) working in these areas. Village assessments were conducted in early 2011 and included questions about village boundaries, population including sex and age, household health and economic situation (eg, common illnesses, use of health services, sources of revenue, employment) and presence of non-governmental organisations (NGOs) and microfinance institutions, availability and access to public services (eg, school, healthcare), experience of conflict, security, village leadership commitment and support for the livestock/animal microfinance intervention. Village commitment included a desire by leaders and rural villagers to raise pigs and rabbits. Congolese team members met with local village chiefs, administrative leadership and respected persons to assess the need for and an interest in a livestock productive asset transfer programme being implemented in partnership with their community. The 10 villages were then selected based on operational feasibility of regularly visiting villages and conducting research; local commitment from village chief and administrators; and information related to the village-level assessments. The RFR intervention and evaluation was initiated 2 years after the PFP intervention and evaluation. The timing of the planned RFR intervention and evaluation matched the timeline for the PFP intervention and evaluation allowing for participant identification, interviews and intervention rollout in harmony with PFP. This meant that adults engaged in the delayed control groups of PFP could engage, if eligible and interested, in the RFR evaluation (figure 1).

### Participant recruitment and eligibility criteria

Recruitment for PFP in the 10 villages started in 2011 with raising awareness about the project through engagement of village leadership and community meetings led by the PFP team. Interested men and women (16 years and older) were eligible to participate in PFP if they expressed an understanding and commitment to the microfinance principles (eg, credit and repayment of loans), were permanent residents of the village, and were responsible individuals in the household. The eligibility criteria of 16 years and older accommodated rural realities where, due to the conflict, elder siblings are responsible for younger family members and some women are married and have children by 16 years of age. From village assessment and meetings with local leaders, our team was aware of the widespread impact of conflict on economic stability, health and relationship between adults in the household and the larger community. Therefore, we did not require an eligibility screen at the household level for individual experiences of traumatic events or level of poverty to participate in PFP. Interested participants were invited to a second meeting to answer any questions about the project, assess eligibility criteria of interested participants and explain and complete the lottery to randomise eligible participants to intervention or delayed control groups. To avoid the perception within the community that certain villagers/households were favoured to receive a pig loan, we conducted a transparent process for randomisation of households (ie, the unit of randomisation) to either intervention or delayed control groups (those receiving the offspring from loan repayment) in the community through a village lottery. Eligible participants placed their name cards in a box and a child from the village randomly selected names representing village households to either the intervention and delayed control groups. This randomisation process of households to intervention or delayed control group was held in each of the 10 villages. For PFP, the team initially planned to enroll 66 households (one adult per household; 33 per group) per village for a total of 660 participants in 10 villages. The high level of interest in PFP as demonstrated by the high number of participants in the community meetings and village lottery led to a shift by the team from restricting the number of households to creating a second delayed control group. Owing to PFP programme model, where the original loan is repaid with 2 piglets, the 33 households randomised to the intervention group in the village will reimburse 66 piglets, which could accommodate loans for additional households in a second delayed control group. A total of 878 male and female adults (16 years and older) were randomly assigned to either intervention or one of the two delayed control groups in the 10 villages.

Recruitment for the RFR impact evaluation started in 2013, 2 years after initiation of PFP. Boys and girls 10–15 years of age were eligible for participation in RFR if they and their parents/caregivers expressed an interest and commitment to the programme. Three groups were planned as part of the comparative effectiveness study; households with eligible adolescents were assigned to: (1) PFP only (ie, intervention with adults only); (2) PFP and RFR (ie, interventions with adults and youth); and (3) RFR only (ie, intervention with youth only). As the RFR evaluation builds off of the sample of households participating in the PFP evaluation, a randomisation exercise was not conducted for RFR. The PFP programme was already on-going in the same villages, therefore the programme representatives (PFP agents) had already established relationships in the community. To create these 3 groups in the 10 villages, all eligible and interested households already enrolled in the PFP delayed control groups (figure 1) were recruited to participate in the RFR intervention and evaluation. Participants from the first PFP delayed control group comprised the adult only group in RFR. Participants from the second PFP delayed control group comprised the adult and adolescent group in RFR. Participants for the RFR adolescent-only group were recruited through outreach to households where there were eligible and interested adults and youth with the help of local leaders. Households that were recruited to this group could not be participating in the PFP impact evaluation, as the adolescent-only group did not include loan of a pig to parent/caregiver. A total of 509 boys and girls ages 10–15 years were assigned to one of three intervention groups.

### Training

The Congolese team composed of five lead staff trained in the livestock productive asset transfer programme by PAIDEK and the implementation and evaluation component of the project by the Johns Hopkins team. The evaluation training focused on research ethics; each staff successfully completed online research ethics training programme provided by FHI 360. The training included building skills in data collection, management and storage of data on tablets and detailed reviews of the evaluation questionnaire and interview techniques including providing support through referrals. Congolese interviewers participated in classroom training, mock interviews and pilot interviews with men and women in the PFP demonstration site. Pilot interviews helped determine the length of interview, identify areas of difficulty and lack of clarity in questions by the staff and participants. During the pilot testing, despite sensitive questions (eg, sexual assault, trauma, IPV), male and female rural villagers expressed comfort whether interviewed by a male or female staff member. The lead staff with support by the Hopkins team in turn trained 10 additional male and female interviewers using the same curriculum to support the fieldwork required to complete the evaluation for both PFP and RFR.

### Study instruments

The evaluation questionnaires for both PFP and RFR were developed after a review of existing and validated tools that have been used in similar low-resource and conflict-affected settings and through multiple pilot field tests with adults and young adolescents in the PFP demonstration sites. Questionnaire refinement was an iterative process that lasted several months. Meetings between the Congolese and US team members and including local Congolese experts in physical and mental health reviewed all items for relevance, cultural meaning and acceptability by the community. After development of a tablet-based questionnaire for use in one-on-one interview, the modified interviews were piloted in rural villages and modified based on feedback. The final questionnaires were translated and back translated from English to French and from French to local languages (Swahili, Mashi). The PFP questionnaire includes assessment of household sociodemographic status, economic stability and employment, past experiences of violence, mental and physical health, family relationships, partner violence, household and village security, life satisfaction and stigma. Table 2 and Table 3 provide a summary of the standardised measures that were adapted for use in the PFP and RFR assessments respectively.

**Table 2:** Summary of adapted and standardised scales in the Pigs for Peace impact evaluation*

Key indicators	Instrument	Description
Household food security	FANTA III/USAID Household food insecurity access scale (past month)^29^ and Household Dietary Diversity Score (past day)^30^	Questions on household food security in the past month and consumption in the past day
General health and function	SF-8 Health Survey^26^	8 questions on physical and emotional health in the past 4 weeks
Past experience of trauma	Harvard Trauma Questionnaire^31^	Participant ever experienced 18 different types of trauma
Mental health	Hopkins Symptom Checklist^32^	25 questions on symptoms of anxiety and depression in the past 4 weeks
Trauma symptoms	Harvard Trauma Questionnaire^31^	16 questions on trauma symptoms experienced in the past week
Intimate partner violence	Modified Conflict Tactics Scale^33^ ^34^	Ever and past year experience of specific acts of physical, psychological and sexual intimate partner violence
Stigma	Devaluation—Discrimination Scale; Adapted Negative Self-Perception Scale;^35^ Everyday Discrimination Scale^36^	Measured (1) perceived stigma, (2) internalised stigma, (3) experienced stigma

*In addition to the measures listed in the table, questions to assess sociodemographic health, economic security and employment, use of healthcare services, household and village security, family relationships were developed for this assessment.

SF-8, Short Form 8-item Health Survey.

**Table 3:** Summary of adapted and standardised scales in the Rabbits for Resilience impact evaluation*

Key indicators	Instrument	Additional information
Household food security	FANTA III/USAID Household food insecurity access scale (past month)^29^ and Household Dietary Diversity Score (past day)^30^	Questions on household food security in the past month and consumption in the past day
Past experience of trauma	Harvard Trauma Questionnaire^31^	Participant ever experienced 18 different types of trauma
Psychosocial assessment	Reduced Acholi Psychosocial Assessment Instrument^27^	Experience of 40 different symptoms in the past 7 days using an instrument adapted for rural, postconflict Northern Uganda
Coping skills	Kidcope^37^	Assessed adolescent coping strategies using 22 questions on different strategies (and helpfulness of each strategy) after exposure to a traumatic event, adapted for use with young adolescents in DRC
Self-esteem	Rosenberg Self-Esteem Scale^38^	10 agree/disagree questions on self-esteem
Hope scale	Doucette and Bickman's Life Satisfaction/Hopefulness scale^39^	The scale was adapted in Rwanda and now for Congo. It includes 10 questions on Hope in the past 30 days
Empathy	Adapted Bryant's Empathy Index^40^	10 questions on participant experience of empathy in different situations
Stigma	Devaluation—Discrimination Scale; Adapted Negative Self Perception Scale;^35^ Everyday Discrimination Scale^36^	Measured (1) perceived stigma, (2) internalised stigma, (3) experienced stigma

*In addition to the measures listed in the table, questions to assess sociodemographic health, economic, school attendance social capital, cohesion and social inclusion, conflict and violence, and empathy.

DRC, Democratic Republic of Congo.

### Data collection

The PFP and RFR baseline interviews took place after recruitment and randomisation but prior to microfinance programme training that included details on health, nutrition and well-being of the pig/rabbit, pen/cage for animals, composting, loan distribution and loan repayment. All evaluation-related interviews take place in a private setting of the participant's choice, most often their home. Interviews are initiated after voluntary, informed oral consent and assent, respectively from adults and young adolescents. Adults and young adolescents are informed that their refusal to participate or stop/withdraw from the interview would not affect their access to the livestock/animal microfinance programme. Trained staff using a tablet computer conducts interviews. Use of the tablet is beneficial in multiple ways: (1) reduced logistical burden of printing and managing the questionnaires; and (2) real-time access to the data to monitor data quality and identification of issues so that they could be remedied between interviews. Additionally, rural villagers express more confidence and comfort when answering questions with the use of the tablet computer as compared to paper-based interviews where staff write down responses. Data recorded on tablet are encrypted; once uploaded to a central US-based server, the data are automatically erased from the tablet.

**Figure 2:**
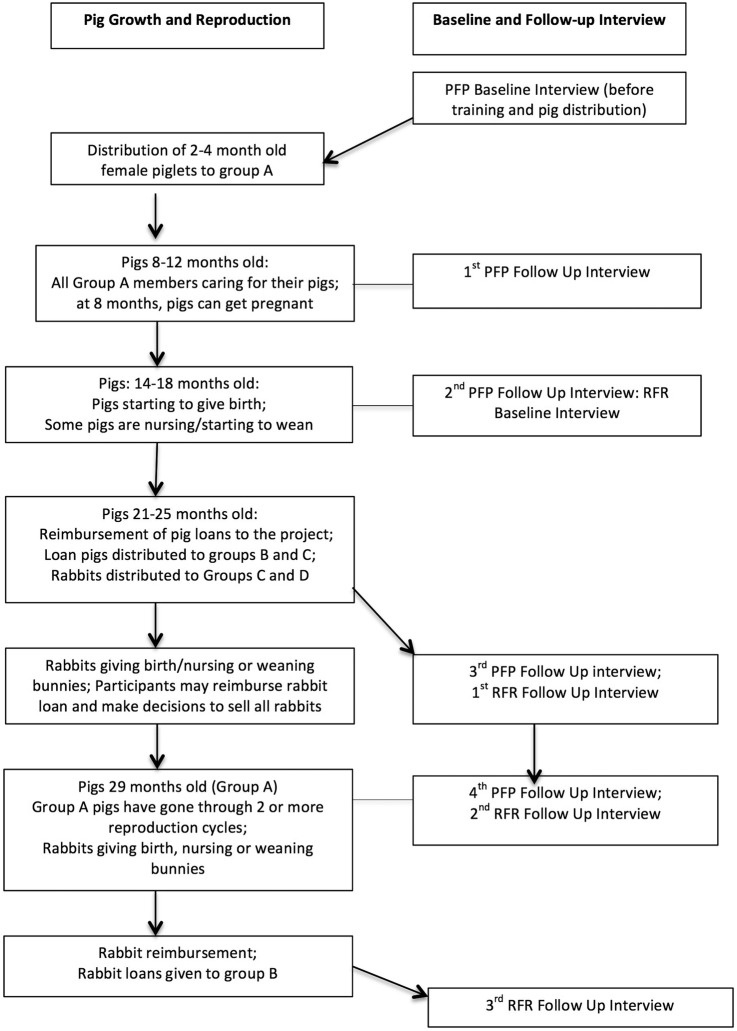
Timing of follow-up interviews linked to pig and rabbit growth and reproduction. PFP, Pigs for Peace; RFR, Rabbits for Resilience.

The frequency of follow-up data collection is based on pig reproduction (birth of offspring) and reimbursement of the animal loan (figure 2). The strategy, designed during the Congolese-US meetings, captures intervention milestones and reduces the burden of interviews on participants. Over a 5-year period, five interviews (baseline and four follow-ups) will be conducted with participants in PFP and four interviews (baseline, three follow-up) with RFR participants. As participants in RFR adult only and combined adult and adolescent microfinance are also members of PFP, the RFR interviews are connected to the timing of the PFP interview starting with the second follow-up interview. Timing of data collection for the follow-up interviews are connected with pig growth and development (1st PFP follow-up); pig mating and initial reproduction (2nd PFP follow-up; RFR baseline); reimbursement and distribution of pig loan to the two delayed control groups and distribution of rabbit loan to intervention groups (3rd PFP follow-up; 1st RFR follow-up); complete pig loan reimbursement in intervention group and rabbit reimbursement (4th PFP follow-up; 2nd RFR follow-up); distribution of rabbits to delayed control groups (3rd RFR follow-up). At each time point, all interviews within a village are completed during the planned time frame.

### Primary outcomes

The primary outcome of the PFP evaluation is the change in baseline mental health distress at 18 months in the intervention compared to control groups. The primary outcome for the RFR evaluation is the change in baseline mental health distress at 18 months based on self-report from adults and young adolescents. Both the PFP evaluation and RFR evaluation examine the additional effects of the intervention on economic stability and family and community relationships as described in secondary outcomes and in table 1.

### Secondary outcomes

Secondary outcomes of interest for the PFP evaluation include household economic stability and improved relationships (eg, reintegration, stigma). The RFR evaluation includes adolescent (eg, school attendance, relationship with family members and peers, self-esteem, outlook for the future), family (eg, household economic stability, food security, parent/caregiver health) and community (youth and adult engagement in activities) resilience as secondary outcomes.

### Sample size and power

Power for PFP is based on a sample size of 300 per group and assumes no change from baseline to 18 months in the control group, a 10% improvement in the intervention group and an α level of 0.05. We do not have a prior estimate of the intraclass correlation coefficient (ICC) (people nested within villages) so power is estimated for varying ICCs. Power to detect a significant group by time interaction for the physical health is 0.94, 0.89 and 0.83 and mental health component score of the Short Form-8 item Health Survey^26^ is 0.91, 0.84 and 0.78 for ICCs of 0.001, 0.005 and 0.010, respectively. Power for RFR is based on a sample size of 480 children (160 per group), power of 0.80 and α level of 0.05. The study can detect a significant difference between approaches if the change over time In Acholi Psychosocial Assessment Instrument^27^ scores is 2.67, 2.82 and 2.98 greater in one approach for ICCs of 0.001, 0.005 and 0.01, respectively.

### Data analysis

Study hypotheses for PFP and RFR will be tested using intent-to-treat model. Multilevel modelling will be used to determine the effectiveness of village-led microfinance programme, PFP, on (1) participants general health, psychological health (depression, post-traumatic stress disorder, suicidality) and reintegration (stigma, reintegration/acceptance by family) and (2) household (food security, children in school, usage of health services) economic stability in intervention villages compared to delayed control villages. Time (baseline to 18 months postintervention) will form the first level of the model. The second level of the model will be gender for the individual outcomes and household for family-level outcomes.

For RFR, multilevel modelling with robust SEs will be used to account for the nested design. Logistic or normal regression models will be used to assess intervention effectiveness depending on the outcome (ie, adolescent and family resilience outcomes such as reduced mental health distress, increased household economic stability and improved relationship with family members and peers). Multilevel modelling will be used to assess change over time from baseline to 18 months postbaseline in young adolescent and adult community resilience (ie, youth and adult engagement in activities). Finally, separate mediation analyses following the approach proposed by Bauer *et al*^28^ will be run to assess whether changes in adolescent resilience (self-esteem, outlook for the future) mediate the relationship between adolescent participation in microfinance and adolescent outcomes (mental health, relationships with family and peers).

## Ethics and dissemination

### Ethical approval

In addition, a committee of respected educators at Universite Catholique de Bukavu and community members (due to lack of local ethics review board) reviewed and approved the study including risks and benefits to participants. Pilot and study interviews were initiated only after receiving oral, voluntary informed consent from the participant for PFP and oral, voluntary informed assent and consent from adolescents and their parents/caregivers, respectively, in RFR. Oral consent was approved during ethics review as the majority of our participants had never attended school, so written consent was a significant challenge. Study identification codes and names were recorded during one-on-one interview; all data recorded through the tablet-based programme was uploaded to a password-protected server managed by the study team. Names were centrally removed and stored in a separate file. As interviews were conducted during the day when members would be earning their daily income, compensation (∼US$1.50) for the time (∼45–90 min) spent away from work was provided.

## Discussion

Findings from these two longitudinal, community trials will provide essential information on effective and sustainable interventions to improve health, economic stability and relationships in rural, postconflict communities. Limited global resources, multiple competing demands for humanitarian and development programmes (eg, health, infrastructure, employment, education, nutrition) in postconflict settings and the growing population living with insecurity requires effective and sustainable models of intervention. The PFP and RFR programmes are developed for and adapted to the needs of rural communities that have experienced multiple traumatic events and have limited economic opportunities to acquire wealth and stability. Findings have the potential to provide important information for practitioners and donors on sustainable and culturally relevant and acceptable interventions to mitigate rural poor populations exposure to economic crises (by building traditional savings in the form of livestock) and while reinforcing family and social bonds and improving health. Further, the RFR evaluation will contribute new and important research on the relative impact of combined adolescent and adult livestock productive asset transfer compared to targeted interventions for adolescents or adults separately.

### Limitations

Our studies have limitations. Participants in PFP and RFR were not selected at random for participation. As a result, data are not representative of the village, but instead participants self-selected to participate and then were randomised into the programmes. While randomisation of all eligible village residents to intervention and delayed control groups was considered, this was not feasible as not all families in participating villages wanted to raise pigs or rabbits in their households. Second, for PFP, although multiple efforts were made to explain the principles of microfinance loans (including loan repayment) to participants, some members may have joined the programme initially expecting the pig loans to be a gift/donation from a humanitarian organisation. Third, increased resources and reduced logistical challenges such as limited infrastructure (eg, well-paved roads) to reach villages during the rainy season and security in rural areas would have allowed the PFP/RFR team members to spend more time in the villages during the start-up of the projects to address early challenges and increase understanding of the microfinance model.

### Dissemination

Study findings will be shared and discussed with DRC and global policymakers and NGOs to advance this sustainable development approach for scale-up in diverse regions of DRC as well as partner with colleagues in other humanitarian settings working to rebuild communities and advance development postconflict. In addition, the findings will be disseminated through local partnerships and workshops in the Great Lakes region of Africa and through publication in peer-review journals. Findings will also be shared in conference abstracts and presentations, workshops globally in collaboration with our Congolese team.

### Conclusion

The findings will provide important information on the potential for community-led sustainable development initiatives to build on traditional livelihood (livestock raising, agriculture) assets to have a sustained health, economic and social impact on the individual, family and community. Further, the design and methods of intervention and evaluation are useful to other microfinance and asset transfer programme staff, researchers and donor organisations to replicate and scale-up similar development projects in diverse rural and potentially urban areas globally.
